# Simultaneous measurements of solid–liquid interfaces and bulk liquids using soft X-ray absorption spectroscopy

**DOI:** 10.1107/S1600577526004637

**Published:** 2026-06-01

**Authors:** Fumitoshi Kumaki, Masanari Nagasaka

**Affiliations:** a Photon Factory, Institute of Materials Structure Science High Energy Accelerator Research Organization 1-1 Oho Tsukuba Ibaraki305-0801 Japan; b Department of Chemistry Keio University 3-14-1 Hiyoshi Yokohama223-8522 Japan; c Institute for Molecular Science Myodaiji Okazaki444-8585 Japan; d Graduate Institute for Advanced Studies, SOKENDAI, Myodaiji, Okazaki444-8585, Japan; Forschungszentrum Jülich, Germany

**Keywords:** soft X-ray absorption spectroscopy, solid–liquid interface, bulk liquid, simultaneous measurement method

## Abstract

Simultaneous measurement methods for soft X-ray absorption spectroscopy (XAS) of solid–liquid interfaces and bulk liquids have been developed. The O *K*-edge XAS spectra of H_2_O/Au interfaces and bulk H_2_O were measured using a transmission-type liquid cell, in which the liquid layer was sandwiched between Au/Cr/Si_3_N_4_ and Si_3_N_4_ membranes. The XAS spectrum of bulk H_2_O was obtained using the transmission method by controlling the thickness of the liquid layer, whereas the XAS spectrum of the H_2_O/Au interface was obtained using the electron-yield method by measuring the drain currents from the Au surface after soft X-ray absorption. The XAS spectra of bulk H_2_O and the H_2_O/Au interfaces were differentiated to determine the appropriate measurement conditions for the solid–liquid interfaces interfaces.

## Abstract 2

Simultaneous measurement methods have been developed for soft X-ray absorption spectroscopy of solid–liquid interfaces using the electron-yield method and bulk liquids using the transmission method.

## 1. Introduction

Water molecules at solid–liquid interfaces play important roles in various catalytic, electrochemical and biological reactions (Björneholm *et al.*, 2016 ▸; Shimizu *et al.*, 2018 ▸; Hu *et al.*, 2024 ▸; Qiu *et al.*, 2025 ▸). Recently, several measurement methods have been developed for observing solid–liquid interfaces. The interface between liquid water and a silicon thin film has been measured using X-ray photoelectron spectroscopy (XPS) (Masuda *et al.*, 2013 ▸). The structure of water molecules on electrode surfaces has been investigated using ambient-pressure XPS (Favaro *et al.*, 2016 ▸) and infrared spectroscopy (Ashley & Pons, 1988 ▸; Nakamura *et al.*, 2008 ▸). The adsorption structures of water molecules at solid–liquid interfaces have been studied using sum-frequency generation spectroscopy (Nihonyanagi *et al.*, 2004 ▸; Tabassum *et al.*, 2025 ▸), Raman spectroscopy (Pettinger *et al.*, 1981 ▸; Wang *et al.*, 2021 ▸) and X-ray scattering (Toney *et al.*, 1994 ▸). The alignment of water molecules on biological membranes has been investigated using surface-specific vibrational spectroscopy (Dreier *et al.*, 2018 ▸). The electronic structures of solid–liquid interfaces during various electrocatalytic reactions have been extensively studied using X-ray absorption spectroscopy (XAS) in both the hard and soft X-ray regions (Timoshenko & Cuenya, 2021 ▸).

Soft X-rays below 2 keV include the *K*-edges of C, N, O and F, and soft X-ray XAS is an element-specific method for investigating the electronic structures of liquid water and organic molecules (Stöhr, 1992 ▸). However, XAS measurements of liquids are difficult to obtain in transmission mode owing to the thickness of the liquid layer, which should be below 1 µm owing to the strong absorption of soft X-rays by air and liquids (Chantler, 2000 ▸). Recently, however, the development of several detection methods has enabled XAS measurements of liquids and solutions (Smith & Saykally, 2017 ▸). Because soft X-ray transmission to liquid samples is challenging, XAS measurements have been performed to detect fluorescent X-rays (Myneni *et al.*, 2002 ▸; Odelius *et al.*, 2006 ▸; Tokushima *et al.*, 2008 ▸) or Auger electrons (Wilson *et al.*, 2001 ▸; Smith *et al.*, 2004 ▸), which are formed as secondary processes after soft X-ray absorption. XAS spectra of liquids have been measured via the fluorescence-yield method using a liquid cell consisting of a Si_3_N_4_ membrane, where soft X-rays irradiate the liquids through the membrane and fluorescence X-rays are emitted from the liquids through the same membrane. However, the XAS spectra in the fluorescence yield deviate from the true absorption spectra because fluorescent X-rays are absorbed by liquid samples, a phenomenon known as the self-absorption effect. The XAS spectra of liquids have been measured in electron-yield mode by detecting the Auger electrons emitted by the soft X-ray absorption of liquid microjets under vacuum. Pressure and temperature should be compensated for in microjets under vacuum because these phases differ from those of liquid under atmospheric conditions. The XAS spectra of liquids have been measured by detecting ion currents and exhibited bulk-sensitive electronic structures (Schön, Golnak *et al.*, 2017 ▸; Schön, Xiao *et al.*, 2017 ▸; Ren *et al.*, 2019 ▸). Further evaluation of the XAS spectra in ion-yield mode is necessary because ions in solution dissipate before they reach the detector.

The transmission method is effective for obtaining the true absorption spectra of bulk liquids. Recently, XAS spectra of thin layers of bulk liquids with a thickness of less than several micrometres in flat microjets were measured in transmission mode (Ekimova *et al.*, 2015 ▸; Fondell *et al.*, 2017 ▸; Koralek *et al.*, 2018 ▸; Kubin *et al.*, 2018 ▸). Although flat microjets show no radiation damage to liquid samples owing to the windowless system, they cannot be applied to liquid samples with high vapor pressures. A transmission-type liquid cell consisting of two Si_3_N_4_ or SiC membranes can be applied to all liquid samples (Yang & Kirz, 1987 ▸; Schreck *et al.*, 2011 ▸; Meibohm *et al.*, 2014 ▸; Sellberg *et al.*, 2014 ▸). XAS spectra of solutions in a wide concentration range were measured by precisely controlling the thickness of the liquid layers (Nagasaka *et al.*, 2020 ▸; Nagasaka & Kosugi, 2021 ▸). Liquid cells can precisely control chemical environments, such as the liquid temperature (Nagasaka *et al.*, 2017 ▸) and electrode potential, using a gold-deposited Si_3_N_4_ membrane as a working electrode (Nagasaka *et al.*, 2013 ▸; Nagasaka, Yuzawa *et al.*, 2014 ▸).

XAS spectra acquired in transmission mode are mainly derived from bulk liquids, and the contributions of the solid–liquid interfaces are smaller than those of bulk liquids. The XAS spectra of solid–liquid interfaces have been frequently obtained by detecting Auger electrons, which mostly originate from solid–liquid interfaces owing to the low escape depth of electrons (Velasco-Velez *et al.*, 2014 ▸; Wu *et al.*, 2018 ▸; Kao *et al.*, 2020 ▸; van Spronsen *et al.*, 2021 ▸; Wang *et al.*, 2025 ▸). The XAS spectra of solid–liquid interfaces with induced electrode potentials have been measured using the electron-yield method (Velasco-Velez *et al.*, 2014 ▸; Wu *et al.*, 2018 ▸; Kao *et al.*, 2020 ▸; van Spronsen *et al.*, 2021 ▸). The hydrogen bonds in bulk H_2_O have been extensively investigated using O *K*-edge XAS (Smith *et al.*, 2004 ▸; Wernet *et al.*, 2004 ▸; Fransson *et al.*, 2016 ▸; Nagasaka *et al.*, 2017 ▸; Frati *et al.*, 2020 ▸). The pre-edge peak in bulk H_2_O reflects the electronic structures of the acceptor sites in H_2_O because it corresponds to the transition of O 1*s* electrons to 4*a*_1_ unoccupied orbitals that are distributed around oxygen atoms. Valesco-Velez *et al.* measured the O *K*-edge XAS spectra of bulk H_2_O in fluorescence-yield mode, and those of the H_2_O/Au interfaces in electron-yield mode (Velasco-Velez *et al.*, 2014 ▸). The pre-edge peak intensity of the H_2_O/Au interface is smaller than that of bulk H_2_O and varies with electrode potentials. By contrast, the pre-edge peak intensities of isolated H_2_O molecules in aqueous aceto­nitrile solutions are stronger than those of bulk H_2_O (Nagasaka, 2024 ▸). Wang *et al.* measured the O *K*-edge XAS spectra of bulk H_2_O using the ion yield and those of H_2_O/Au interfaces using the electron yield, finding that the pre-edge peaks of the H_2_O/Au interfaces are also weaker than those of bulk H_2_O (Wang *et al.*, 2025 ▸).

**Figure 1:**
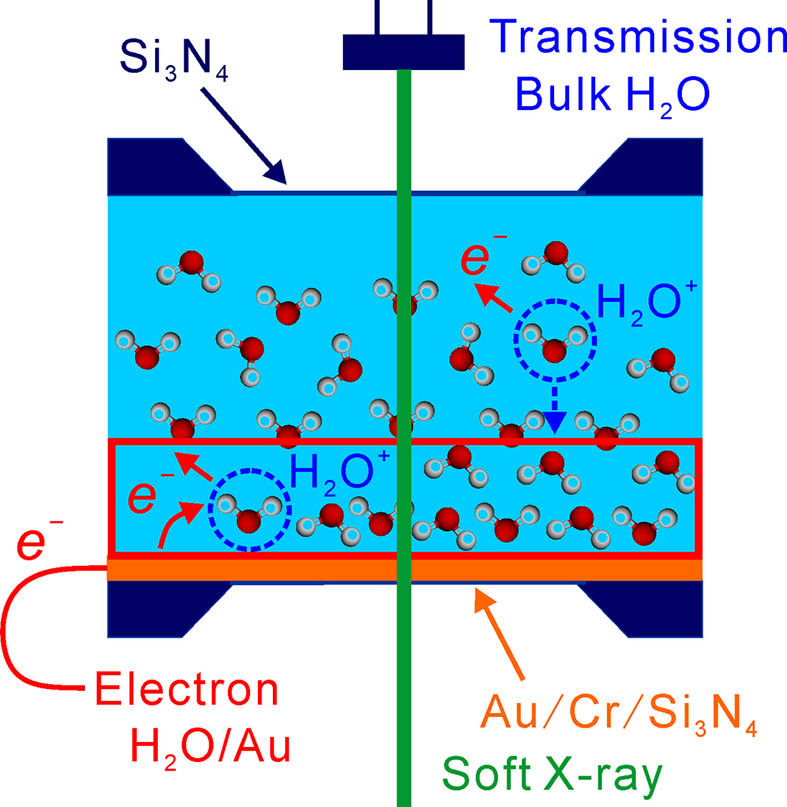
Schematic of simultaneous XAS measurements for H_2_O/Au interfaces and bulk H_2_O. The H_2_O/Au interfaces were measured using the electron-yield method, and bulk H_2_O was measured using the transmission method. The excitation processes of H_2_O molecules after soft X-ray absorption at the interface and bulk liquid are also described.

As described above, the XAS spectra of the solid–liquid interfaces were measured using the electron-yield method, and those of bulk liquids were measured using fluorescence or ion yield methods. As shown in Fig. 1 ▸, this study developed simultaneous XAS measurements of bulk H_2_O and H_2_O/Au interfaces by applying the electron-yield method using a transmission-type liquid cell, including an Au/Cr/Si_3_N_4_ membrane. Because the XAS spectra in transmission mode reflect the true absorption spectra of bulk H_2_O, the differences between bulk H_2_O and H_2_O/Au interfaces can be accurately determined. The electron-yield XAS spectra were obtained by measuring drain currents from the Au/Cr/Si_3_N_4_ membrane to compensate electrons for H_2_O^+^ cations, which are formed by emitting Auger electrons after soft X-ray absorption. Because the effective attenuation length of electrons in liquid water is 6 nm at the O *K*-edge (Suzuki *et al.*, 2014 ▸), most of the electron-yield XAS spectrum is derived from the H_2_O/Au interfaces. Although H_2_O^+^ cations are also formed in bulk H_2_O by emitting Auger electrons after soft X-ray absorption, there is few amount of electrons from the Au/Cr/Si_3_N_4_ membrane due to long distances. Note that a small amount of H_2_O^+^ cations in bulk H_2_O reaches the Au/Cr/Si_3_N_4_ membrane owing to the diffusion or exchange of H_2_O^+^ cations because the XAS spectra of liquid H_2_O were obtained by the ion-yield measurements (Wang *et al.*, 2025 ▸). Thus, a small drain current is formed for the H_2_O^+^ cations come from bulk H_2_O. This study explores the appropriate measurement conditions for H_2_O/Au interfaces using electron-yield XAS measurements.

## 2. Experimental setup

**Figure 2:**
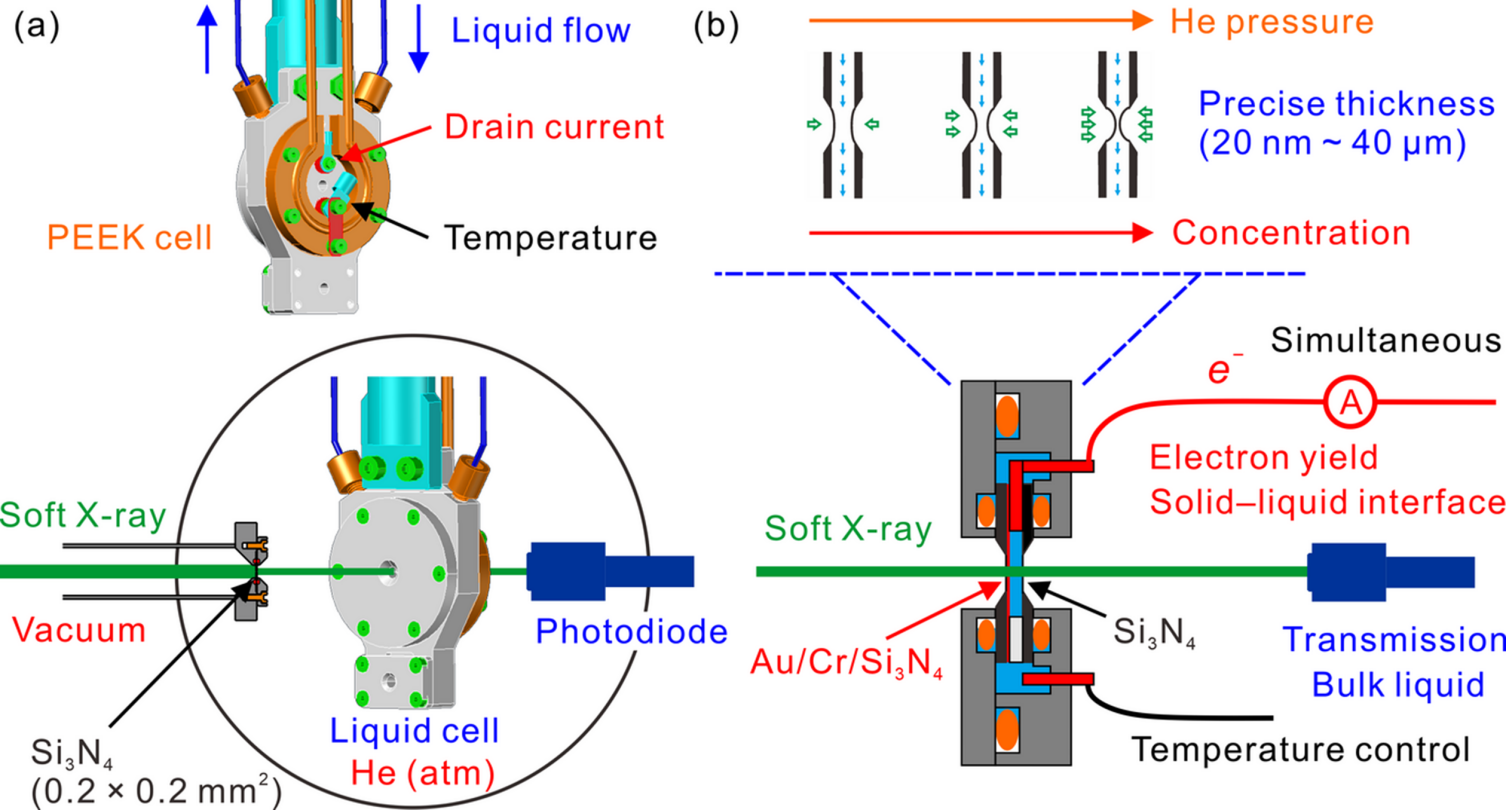
(*a*) Schematic of the simultaneous XAS measurement method for solid–liquid interfaces and bulk liquids. The back side of the liquid cell is also shown. (*b*) Cross-sectional view of the liquid cell, where solid–liquid interfaces were measured using the electron-yield method and bulk liquids were measured using the transmission method. A schematic of the precise thickness control of the liquid layer from 20 nm to 40 µm for the XAS measurements in transmission mode is also shown.

Fig. 2 ▸(*a*) shows schematics of the simultaneous XAS measurement system for solid–liquid interfaces and bulk liquids. The experiments were performed at the soft X-ray beamline BL-13A at the Photon Factory, Institute of Materials Structure Science, High Energy Accelerator Research Organization (KEK-PF), Japan (Mase *et al.*, 2010 ▸; Toyoshima *et al.*, 2013 ▸). Details of the transmission-type liquid cells used for the XAS measurements are provided elsewhere (Nagasaka *et al.*, 2018 ▸; Nagasaka *et al.*, 2020 ▸; Nagasaka & Kosugi, 2021 ▸). The liquid cell was set in an atmospheric helium chamber, which was separated by a 100 nm-thick Si_3_N_4_ membrane with a window size of 0.2 mm × 0.2 mm (NTT-AT) from the soft X-ray beamline under ultrahigh vacuum. The liquid layer in the liquid cell consisted of two membranes with a window size of 2 mm × 2 mm: a 100 nm-thick Si_3_N_4_ membrane and an Au (20 nm)/Cr (5 nm)/Si_3_N_4_ (100 nm) membrane. Transmission measurements were performed by detecting the transmitted soft X-rays using a photodiode detector (Opto Diode IRD AXUV 100). The XAS spectrum of bulk H_2_O was obtained using the Beer–Lambert law, ln(*I*_0_/*I*), where *I* is the transmission signal of bulk H_2_O and the membranes, and *I*_0_ is the transmission signal of the membranes.

The liquid cell was composed of polyether ether ketone (PEEK) resin to provide electrical insulation of the entire system during the electron-yield measurements. The back side of the liquid cell was equipped with terminals for measuring drain currents caused by soft X-ray irradiation and for controlling the liquid temperature. Liquid samples were exchanged using a syringe pump. The liquid cell, consisting of stainless-steel plates, can control the liquid temperature from −5 to 80°C using a temperature-controlled Cu plate with a chiller system (Nagasaka *et al.*, 2020 ▸). By contrast, the liquid cell with PEEK resin was used only at room temperature owing to the resin’s low thermal conductivity. The developed liquid cell included an Au terminal to control the liquid temperature. The temperature of the liquid layer was adjusted by controlling the temperature of the Au terminal using a chiller. The liquid temperature could be increased to over 200°C using a sheathed heater for catalytic reactions at high temperatures. Note that the Au terminal was electrically isolated from the heaters for the electron-yield measurements. In the present study, the XAS spectra of the solid–liquid interfaces were measured at room temperature without using a thermal control unit.

Fig. 2 ▸(*b*) shows a cross-sectional view of the liquid cell. The liquid layer in the liquid cell was sandwiched between Si_3_N_4_ and Au/Cr/Si_3_N_4_ membranes with window sizes of 2 mm × 2 mm, which were attached to Si frames with sizes of 10 mm × 10 mm. The liquid layer was prepared by setting Teflon and Au spacers to have a thickness of 100 µm at both sides of the Si frames and was tightly sealed using O-rings outside the Si frames. The Si frames in the central parts were close to each other owing to the pressing of the O-rings. The temperature of the liquid layer was controlled using a chiller or heater system through the Au terminal, which was electrically isolated and measured using a Pt100 resistance temperature detector connected to the Au terminal. The XAS spectra of the electron yield were obtained by measuring the drain currents of the Au/Cr/Si_3_N_4_ membrane after soft X-ray absorption through the Au spacer. Electron-yield measurements provided the XAS spectra of the H_2_O/Au interfaces owing to the low escape depth of Auger electrons. The drain currents were converted to voltages using a programmable preamplifier (NF Corporation CA5351), where the signal-to-noise ratio of the drain currents was improved using an electric filter with slow response time. The voltages were converted into TTL signals using a V–F converter (Tsujicon SN2VF-01), and the TTL signals were collected using a TTL counter (Ortec 974).

The XAS spectra of bulk liquids were obtained via transmission measurements. As shown in the inset of Fig. 2 ▸(*b*), the thickness of the liquid layer was precisely controlled from 20 nm to 40 µm by changing the pressure of helium gas around the liquid cell. This method realizes XAS measurements of solutions in wide concentration ranges, where thick liquid layers are prepared for dilute solutions and thin liquid layers are used for condensed solutions. The N *K*-edge XAS spectra of metal complexes in aqueous solutions can be measured at concentrations of several m*M* using the water window spectral region (Nagasaka *et al.*, 2024 ▸). The intensities of the transmitted soft X-rays were measured using a photodiode detector. The currents from the detector were converted to voltages using a programmable preamplifier (NF Corporation CA5350), voltages were converted to TTL signals using the V–F converter, and TTL signals were counted using the TTL counter.

## 3. Application to interfacial water on gold surface

**Figure 3:**
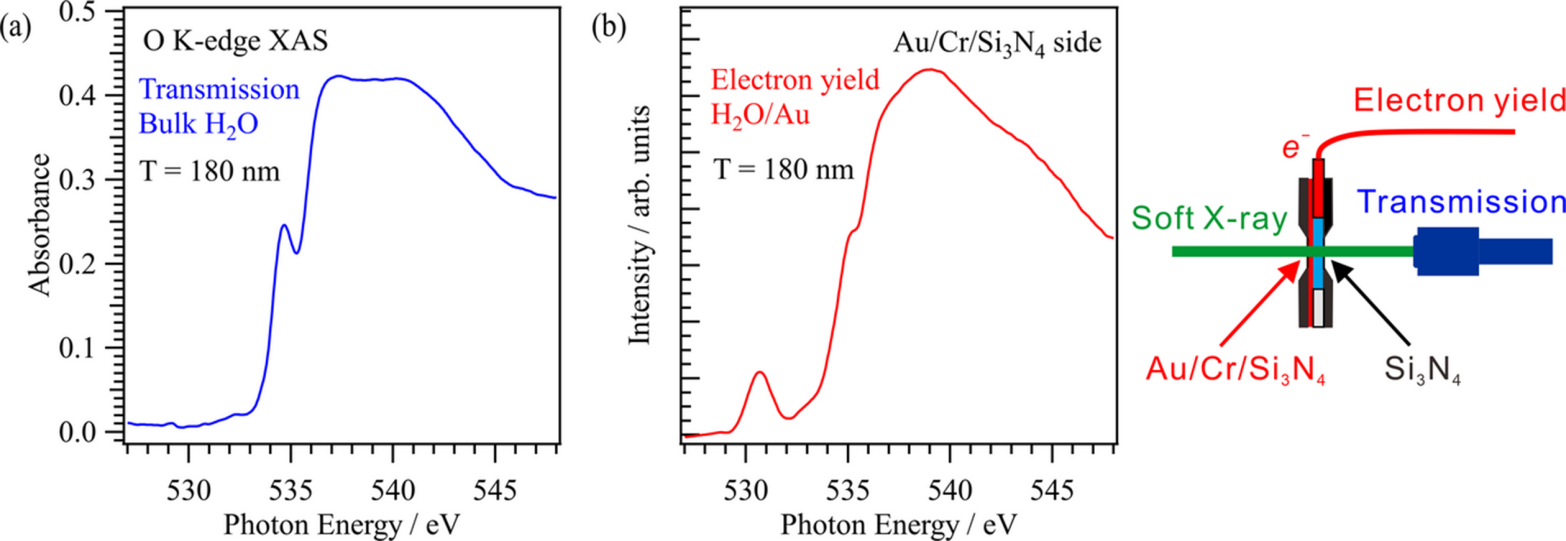
O *K*-edge XAS spectra of liquid H_2_O on the Au/Cr/Si_3_N_4_ membrane. The XAS spectra were measured by the irradiation of soft X-rays to the side of the Au/Cr/Si_3_N_4_ membrane in the liquid cell, as shown in the inset. (*a*) O *K*-edge XAS spectrum of bulk H_2_O obtained using the transmission method. (*b*) O *K*-edge XAS spectrum of the H_2_O/Au interface obtained using the electron-yield method.

Fig. 3 ▸ shows the O *K*-edge XAS spectra of liquid H_2_O on the Au/Cr/Si_3_N_4_ membrane. The spectra of bulk H_2_O were obtained using the transmission method, and those of the H_2_O/Au interfaces were simultaneously obtained using the electron-yield method. The side of the Au/Cr/Si_3_N_4_ membrane in the liquid cell was irradiated with soft X-rays. The O *K*-edge XAS spectrum of bulk H_2_O is consistent with those of previous studies (Smith *et al.*, 2004 ▸; Wernet *et al.*, 2004 ▸; Nagasaka *et al.*, 2017 ▸), where the pre-edge, main-edge and post-edge peaks appear at ∼534.7, ∼537 and ∼540 eV, respectively. The small peak at ∼532 eV is attributed to the oxide layers of the Au/Cr/Si_3_N_4_ and Si_3_N_4_ membranes. The thickness of the liquid layer was estimated to be 180 nm from the edge jump of the XAS spectrum using the calculated absorption coefficient of liquid H_2_O (Chantler, 2000 ▸).

Fig. 3 ▸(*b*) shows the O *K*-edge XAS spectrum of the H_2_O/Au interface, obtained using the electron-yield method. The pre-edge peak at the H_2_O/Au interface merges with the shoulder of the main-edge peak owing to the higher energy shift of the pre-edge peak compared with that of bulk H_2_O. These spectral shapes are consistent with previous XAS measurements obtained using the electron-yield method (Velasco-Velez *et al.*, 2014 ▸; Wang *et al.*, 2025 ▸). The pre-edge peaks in the O *K*-edge XAS spectra of liquid methanol (Nagasaka, Mochizuki *et al.*, 2014 ▸) and liquid ethanol (Nagasaka *et al.*, 2022 ▸) also appear at the shoulder of the main-edge peak because the electronic structures of the OH groups are changed by the methyl and ethyl groups. The pre-edge peaks of the H_2_O/Au interfaces reflect the electronic structures of H_2_O molecules interacting with Au surfaces. The oxide layers of the Au/Cr/Si_3_N_4_ membrane give rise to a sharp peak at ∼530.7 eV. These results confirm that the electronic structures of the solid–liquid interfaces are clearly obtained from the XAS spectra using the electron-yield method.

**Figure 4:**
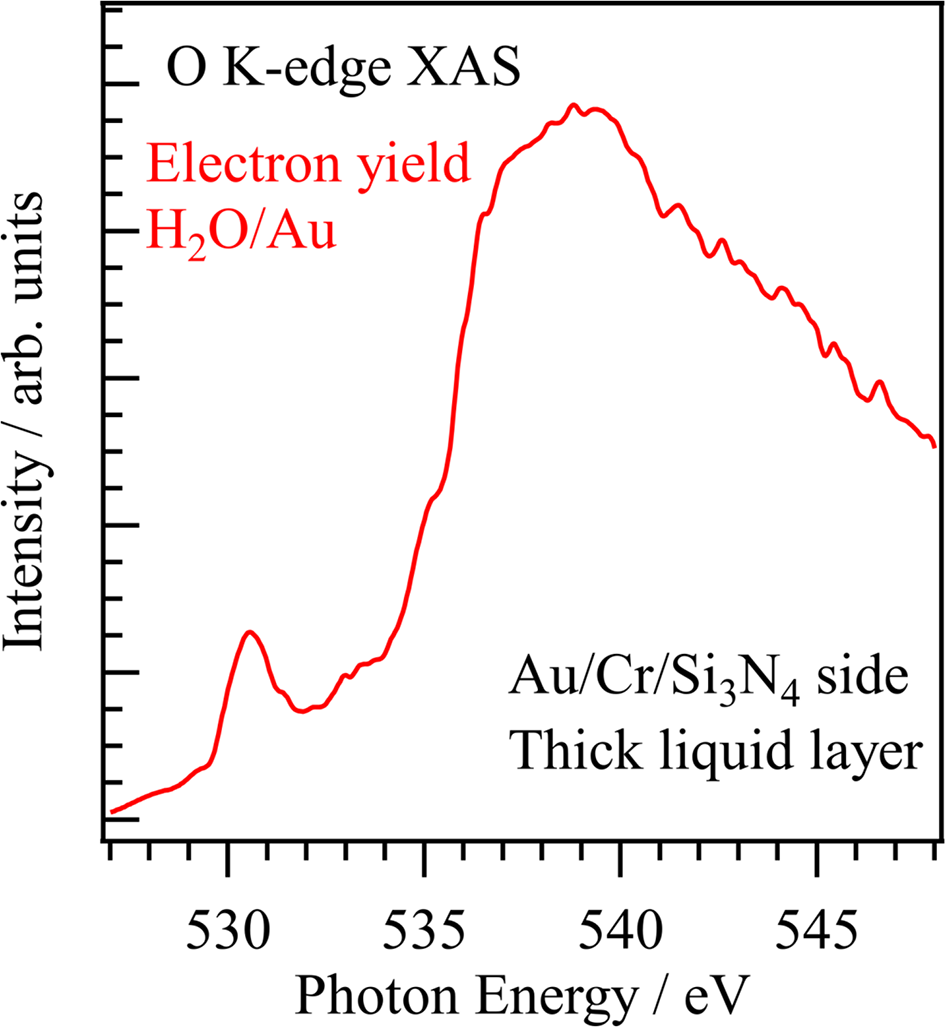
O *K*-edge XAS spectrum of the H_2_O/Au interface for a thick liquid layer obtained via the electron-yield method, where soft X-rays irradiated to the side of the Au/Cr/Si_3_N_4_ membrane.

Fig. 4 ▸ shows the O *K*-edge XAS spectrum of the H_2_O/Au interface for a thick liquid layer, obtained using the electron-yield method. The side of the Au/Cr/Si_3_N_4_ membrane was irradiated with soft X-rays. Although soft X-rays could not be transmitted to the thick liquid layer under the present conditions, the membrane-related oxide peak and pre-edge peak at the H_2_O/Au interfaces were obtained in the electron-yield measurements. In other words, XAS measurements of the solid–liquid interfaces are possible by irradiating the side of the Au/Cr/Si_3_N_4_ membrane with soft X-rays. Note that the electron-yield XAS spectra of H_2_O/Au interfaces would have the same spectral profiles at different thickness of the liquid layer for soft X-rays irradiating the Au/Cr/Si_3_N_4_ side. However, the profile of the electron-yield XAS spectrum shown in Fig. 4 ▸ is slightly different from that shown in Fig. 3 ▸(*b*). This discrepancy is caused by deforming of the Au/Cr/Si_3_N_4_ membrane by varying the thickness of the liquid layer, which would change the soft X-ray irradiation positions.

## 4. Discussion on appropriate measurement conditions of interfaces

**Figure 5:**
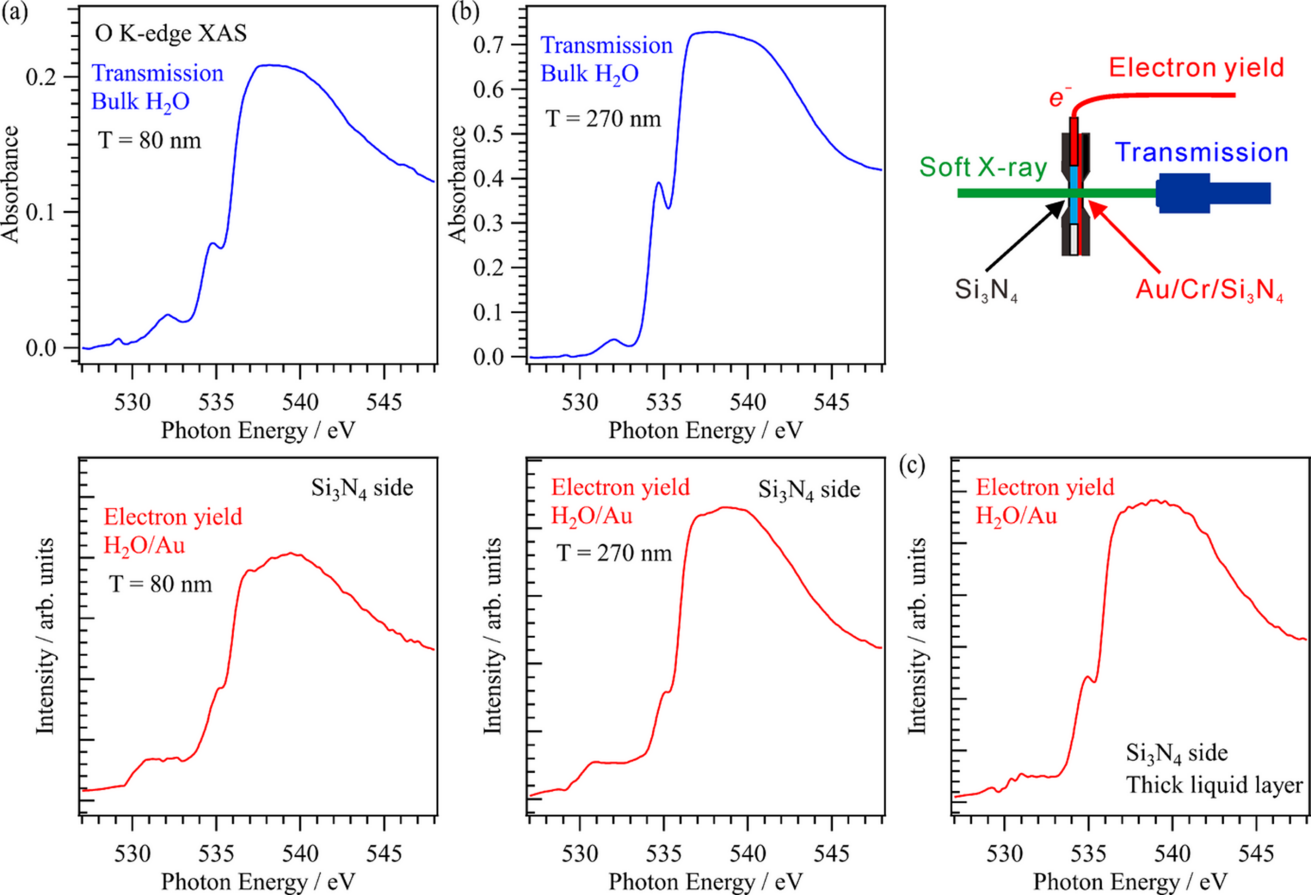
O *K*-edge XAS spectra of bulk H_2_O and H_2_O/Au interfaces at varying thicknesses of the liquid layer, measured by irradiating soft X-rays to the side of the Si_3_N_4_ membrane in the liquid cell (inset). The thicknesses of the liquid layers were (*a*) 80 nm and (*b*) 270 nm. (*c*) Thick liquid layer, where the soft X-rays were strongly absorbed by bulk H_2_O.

For obtaining appropriate measurement conditions of bulk H_2_O and the H_2_O/Au interfaces, the O *K*-edge XAS spectra of liquid H_2_O in transmission mode and the electron yield were measured at different thicknesses of the liquid layer with soft X-ray irradiation to the side of the Si_3_N_4_ membrane. Figs. 5 ▸(*a*) and 5(*b*) show the O *K*-edge XAS spectra of liquid H_2_O with thicknesses of 80 and 270 nm, respectively, measured using the transmission method. The transmission spectra include the contributions of both bulk liquids and solid–liquid interfaces and exhibit a pre-edge peak at ∼534.7 eV and a peak at ∼532 eV derived from the oxide layers of the Au/Cr/Si_3_N_4_ and Si_3_N_4_ membranes. The XAS spectrum at 270 nm thickness is mainly derived from bulk liquids because of the thick layers of bulk H_2_O. In the transmission spectrum at 80 nm thickness, the intensity of the pre-edge peak relative to the main-edge peak is lower than that in the XAS spectrum of the thick liquid layer of bulk H_2_O, indicating that the contribution of the H_2_O/Au interface increases in the XAS spectra of the thin liquid layer. However, the contribution of bulk H_2_O still dominates the XAS spectrum of the thin liquid layer in transmission mode, as indicated by the distinct pre-edge peak of bulk H_2_O. Note that the spectral contributions of bulk H_2_O and the H_2_O/Au interfaces can be separated from the XAS spectra of liquid H_2_O at different thicknesses, where the ratio of bulk H_2_O and H_2_O/Au interfaces varies with different thickness of the liquid layers. In a recent infrared spectroscopy study, the spectrum of vicinal H_2_O on hydro­gels has been separated from that of bulk H_2_O by varying the contribution of vicinal H_2_O and bulk H_2_O (Maeda *et al.*, 2022 ▸).

Fig. 5 ▸ shows the electron-yield XAS spectra of liquid H_2_O at different thicknesses of the liquid layers with soft X-ray irradiation to the side of the Si_3_N_4_ membrane. The electron-yield spectrum at 80 nm thickness is mainly derived from the H_2_O/Au interface, which shows shoulder structures of the pre-edge peak to the main-edge peak, similar to the electron-yield spectrum shown in Fig. 3 ▸(*b*). Note that the membrane-related oxide peak at 532 eV shows a broader profile compared with that shown in Fig. 3 ▸(*b*). The soft X-ray irradiation positions of the membranes in the electron-yield measurements at the Si_3_N_4_ side were different from those at the Au/Cr/Si_3_N_4_ side, and the amount of oxide layers in the irradiation positions would be different from each other. The electron-yield XAS spectrum at 270 nm thickness also shows shoulder structures of the pre-edge peak to the main-edge peak, which is derived from the H_2_O/Au interface. Fig. 5 ▸(*c*) shows the O *K*-edge XAS spectrum of H_2_O/Au interfaces with a thick liquid layer. Because soft X-rays were mostly absorbed by bulk H_2_O before they reached the Au/Cr/Si_3_N_4_ membrane, the membrane-related oxide peak exhibits low intensity. The intensity of the pre-edge peak also increases compared with the main-edge peak, indicating that the ratio of bulk H_2_O increases at the thicker liquid layers.

**Figure 6:**
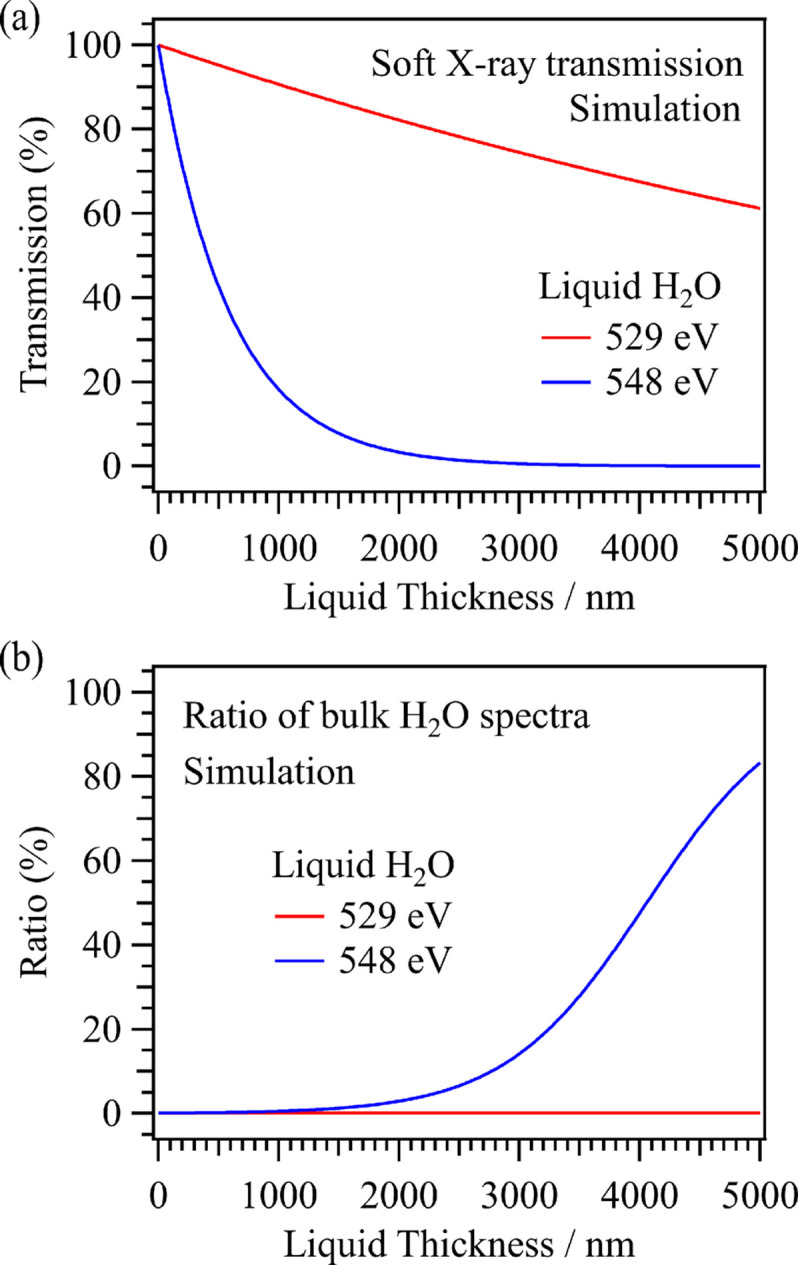
(*a*) Simulated soft X-ray transmission of liquid H_2_O at 529 and 548 eV as a function of the thickness of the liquid layer. (*b*) Ratio of bulk H_2_O spectra at 529 and 548 eV as a function of the thickness of the liquid layer, estimated from the simulated soft X-ray transmission.

The electron-yield XAS spectra of liquid H_2_O at soft X-ray irradiation to the Au/Cr/Si_3_N_4_ side are mostly derived from the H_2_O/Au interface because the effective attenuation length of electrons in liquid H_2_O is 6 nm (Suzuki *et al.*, 2014 ▸). In the electron-yield spectra at soft X-ray irradiation to the Si_3_N_4_ side, on the other hand, the spectral contributions of the H_2_O/Au interface and bulk H_2_O would be changed because soft X-rays were absorbed by bulk H_2_O before they reached the Au/Cr/Si_3_N_4_ membrane. Thus, the influence of the different thicknesses of the liquid layers on the electron-yield XAS spectra was discussed using the simulated soft X-ray transmission of liquid H_2_O. Fig. 6 ▸(*a*) shows the simulated soft X-ray transmission of liquid H_2_O at different thicknesses of the liquid layer, obtained from the calculated soft X-ray absorption coefficients of liquid H_2_O (Chantler, 2000 ▸). Soft X-rays are not strongly absorbed by liquid H_2_O before the O *K*-edge at 529 eV, and the transmission of soft X-rays is 74.5% at 3000 nm thickness. On the other hand, soft X-rays are strongly absorbed by liquid H_2_O after the O *K*-edge at 548 eV, and the transmission of soft X-rays is 0.6% at 3000 nm thickness. This means that the transmission spectra of liquid H_2_O need thin liquid layers below 1000 nm.

When the soft X-ray intensities at the Au/Cr/Si_3_N_4_ membrane decrease with strong absorption of soft X-rays by bulk H_2_O, the ratio of bulk H_2_O spectra caused by the diffusion or exchange of H_2_O^+^ cations becomes enhanced, as shown in Fig. 1 ▸. Thus, the ratio of the H_2_O/Au interface would be reduced by decreasing the soft X-ray intensities at the Au/Cr/Si_3_N_4_ membrane, whereas that of bulk H_2_O would not be changed with the different thickness of the liquid layer. Fig. 6 ▸(*b*) shows the simulated ratio of bulk H_2_O in the electron-yield XAS spectra for different thicknesses of the liquid layer, estimated from the simulated soft X-ray transmission. The ratio of bulk H_2_O was assumed to be 0.1% in the initial condition (0 nm). When the soft X-ray intensity at the Au/Cr/Si_3_N_4_ membrane is reduced by increasing the thickness of the liquid layer, the ratio of bulk H_2_O spectra increases. Because soft X-ray transmission at 529 eV is not strongly changed at 3000 nm thickness, the ratio of bulk H_2_O spectra is not changed from 0.1%. The ratio of bulk H_2_O at 548 eV is not different below 1000 nm thickness. This means that the electron-yield XAS spectra mostly come from the H_2_O/Au interface when the thickness of the liquid layer is below 1000 nm, even if soft X-rays irradiate to the side of the Si_3_N_4_ membrane. Therefore, the electron-yield spectra shown in Figs. 5 ▸(*a*) and 5(*b*) mostly come from the H_2_O/Au interfaces. The ratio of bulk H_2_O spectra at 548 eV increases at the thicker liquid layers and reaches 14.2% at 3000 nm thickness. Therefore, the electron-yield spectrum at the thick liquid layer includes the contribution of bulk H_2_O. Note that the electron-yield XAS spectra at the thick liquid layer would show deformed spectral shapes because soft X-ray absorption of liquid H_2_O at 548 eV is largely different from that at 529 eV. These results suggest that the irradiation of soft X-rays from the Au/Cr/Si_3_N_4_ membrane side is important for clear observation of the H_2_O/Au interface.

## 5. Conclusion

In this study, simultaneous XAS measurement methods for solid–liquid interfaces and bulk liquids were developed using the electron-yield and transmission methods. The liquid layer in the transmission-type liquid cell consisted of Au/Cr/Si_3_N_4_ and Si_3_N_4_ membranes, and the thickness of the liquid layer was precisely controlled from 20 nm to 40 µm (Nagasaka *et al.*, 2020 ▸; Nagasaka & Kosugi, 2021 ▸). The O *K*-edge XAS spectra of bulk H_2_O at varying liquid-layer thicknesses were obtained using the transmission method. The XAS spectra of the H_2_O/Au interfaces were obtained using the electron-yield method, which measures the drain current from the Au surface after soft X-ray absorption. When soft X-rays irradiate the side of the Au/Cr/Si_3_N_4_ membrane in the liquid cell, the XAS spectrum of the H_2_O/Au interface acquired in electron-yield mode exhibits a peak derived from the oxide layer of the membrane, as well as a pre-edge peak of H_2_O molecules that merges with the shoulder of the main-edge peak, which is consistent with previous measurements of H_2_O/Au interfaces (Velasco-Velez *et al.*, 2014 ▸; Wang *et al.*, 2025 ▸). Pre-edge peaks of the H_2_O/Au interfaces in the electron-yield XAS spectra were also obtained with soft X-ray irradiation to the Si_3_N_4_ side when the thickness of the liquid layer was below 1000 nm. The XAS spectra at the thick liquid layer would include the contribution of bulk H_2_O owing to the strong absorption of soft X-rays by liquid H_2_O and would show deformed spectral shapes because the soft X-ray absorption coefficient before the O *K*-edge is largely different from that after the O *K*-edge. These results suggest that the XAS spectra of the H_2_O/Au interfaces and the oxide layer of the membrane should be measured using the electron-yield method with soft X-ray irradiation of the Au/Cr/Si_3_N_4_ membrane side.

The present study investigated H_2_O/Au interfaces using Au/Cr/Si_3_N_4_ membranes. XAS measurements of other interfaces will be applicable by forming thin films on the Au surfaces with the formation of SrTiO_3_ or CoOOH (Wang *et al.*, 2025 ▸), which have been used for catalytic reactions in solutions. Transmission measurements are possible when the catalytic thin films are within several tens of nanometres. Electron-yield measurements are possible when drain currents flow to thin films. Recently, *operando* XAS measurements of electrochemical reactions have been performed using Au/Cr/Si_3_N_4_ membranes as working electrodes (Nagasaka *et al.*, 2013 ▸; Nagasaka, Yuzawa *et al.*, 2014 ▸). Nickel borate electrocatalysts on Au surfaces have been investigated using O *K*-edge XAS (Yoshida *et al.*, 2015 ▸). O *K*-edge XAS measurements also observed a high-valent iron-oxo species, involved in catalytic methane oxidation by the μ-nitrido-bridged iron phthalocyanine dimer deposited on a carbon surface (Yamada *et al.*, 2025 ▸). The electron-yield XAS measurements will be applicable to investigate the interfaces of electrocatalysts during reactions. For biological reactions at solid–liquid interfaces, XAS measurements of membrane proteins can be performed using lipid bilayers including proteins (Goh *et al.*, 2023 ▸). The simultaneous XAS measurement methods for solid–liquid interfaces and bulk liquids are applicable for investigating the mechanisms of various catalytic, electrochemical and biological reactions involving solid–liquid interfaces.

## Data Availability

The data underlying this study are openly available in Figshare at https://doi.org/10.6084/m9.figshare.31330666.
